# Sleep characteristics and HbA1c in patients with type 2 diabetes on glucose-lowering medication

**DOI:** 10.1136/bmjdrc-2020-001702

**Published:** 2020-08-31

**Authors:** Xiao Tan, Christian Benedict

**Affiliations:** Department of Neuroscience, Uppsala Universitet, Uppsala, Sweden

**Keywords:** sleep apnea, obstructive, sleep, diabetes mellitus, type 2

## Abstract

**Introduction:**

To examine the association of sleep duration, insomnia, and obstructive sleep apnea (OSA) with hemoglobin A1c (HbA1c) in a cohort of patients with type 2 diabetes (T2D) on glucose-lowering medications.

**Research design and methods:**

13 346 patients with T2D were included in the present analysis (mean age: 60.2 years; 56.6% were on antidiabetic drug monotherapy; 43.4% received at least two glucose-lowering medications). Sleep duration (short: ≤6 hours/day; normal: 7–8 hours/day; long: ≥9 hours/day) and frequency of insomnia symptoms were self-reported. The risk of OSA was considered high if at least two of the following conditions were fulfilled: regular snoring, frequent daytime sleepiness, and either obesity (≥30 kg/m^2^) or hypertension (systolic blood pressure ≥140 mm Hg or diastolic blood pressure ≥90 mm Hg). Associations between sleep variables and HbA1c were investigated by analysis of covariance or linear regression (adjusted for, eg, participants’ age, sex, ethnic background, and systolic blood pressure).

**Results:**

Long sleep duration and a high risk for OSA were independently associated with higher HbA1c values (long vs normal sleep duration: +0.10% (95% CI 0.03 to 0.18); high vs low risk for OSA: +0.07% (95% CI 0.02 to 0.11), both p=0.004). No robust association was found of short sleep duration and frequent insomnia symptoms with HbA1c. Finally, a positive dose–response association between the number of sleep problems per subject (range: 0–3) and HbA1c was observed (β=0.04% (0.02 to 0.06), p=0.002). However, all significant associations were small.

**Conclusion:**

Screening for and treatment of sleep problems may help lower HbA1c levels in patients with T2D on glucose-lowering medications.

**Significance of this study**

**What is already known about this subject?**

- Epidemiological studies have shown that short and long sleep duration, insomnia, and obstructive sleep apnea (OSA) all correlate with higher hemoglobin A1c (HbA1c) in patients with pre-diabetes and untreated diabetes.

**What are the new findings?**

- Long sleep duration (≥9 hours/day) is associated with higher HbA1c in patients with type 2 diabetes (T2D) on glucose-lowering medications.
- High risk of OSA is associated with higher HbA1c in patients with T2D on glucose-lowering medications.
- Short sleep duration (≤6 hours/day) and frequent insomnia complaints are not or only weakly associated with HbA1c in patients with T2D on glucose-lowering medications.
- All significant associations were small.

**How might these results change the focus of research or clinical practice?**

- Screening for and treatment of sleep problems may help lower HbA1c in patients with T2D on glucose-lowering medications.

## Introduction

Patients with type 2 diabetes (T2D) often chronically suffer from sleep problems, such as insomnia, obstructive sleep apnea (OSA), insufficient sleep duration, and hypersomnia.^1^ This is worrisome as such sleep problems have been linked to impaired glycemic control in patients with T2D.^2–6^ For example, a cross-sectional study involving 52 patients with T2D found that OSA was associated with higher hemoglobin A1c (HbA1c) values.^7^ Long-term elevated blood glucose levels, as measured by the HbA1c level, have also been reported for patients with T2D suffering from poor sleep quality.^8^ Finally, a recent meta-analysis involving 29 649 patients with T2D found that those who reported short (defined as <6 hours) or long (defined as >8 hours) sleep duration exhibited higher HbA1c values.^5^ Whether poor sleep patterns are linked with higher blood glucose levels among patients with T2D on glucose-lowering medications, such as metformin, has however not been systematically investigated.

In the present study involving 13 346 patients with T2D on antidiabetic medications, we therefore investigated the association of sleep duration, OSA, and insomnia with HbA1c. Data were derived from the UK Biobank baseline investigation. Given that many patients with T2D experience multiple sleep problems,^1^ we also investigated whether a dose–response relationship exists between the number of sleep problems per subject and HbA1c. Overall, we hypothesized that sleep problems would be associated with higher HbA1c values in patients with T2D on glucose-lowering medications.

## Methods

### Study design and participants

**Figure 1:**
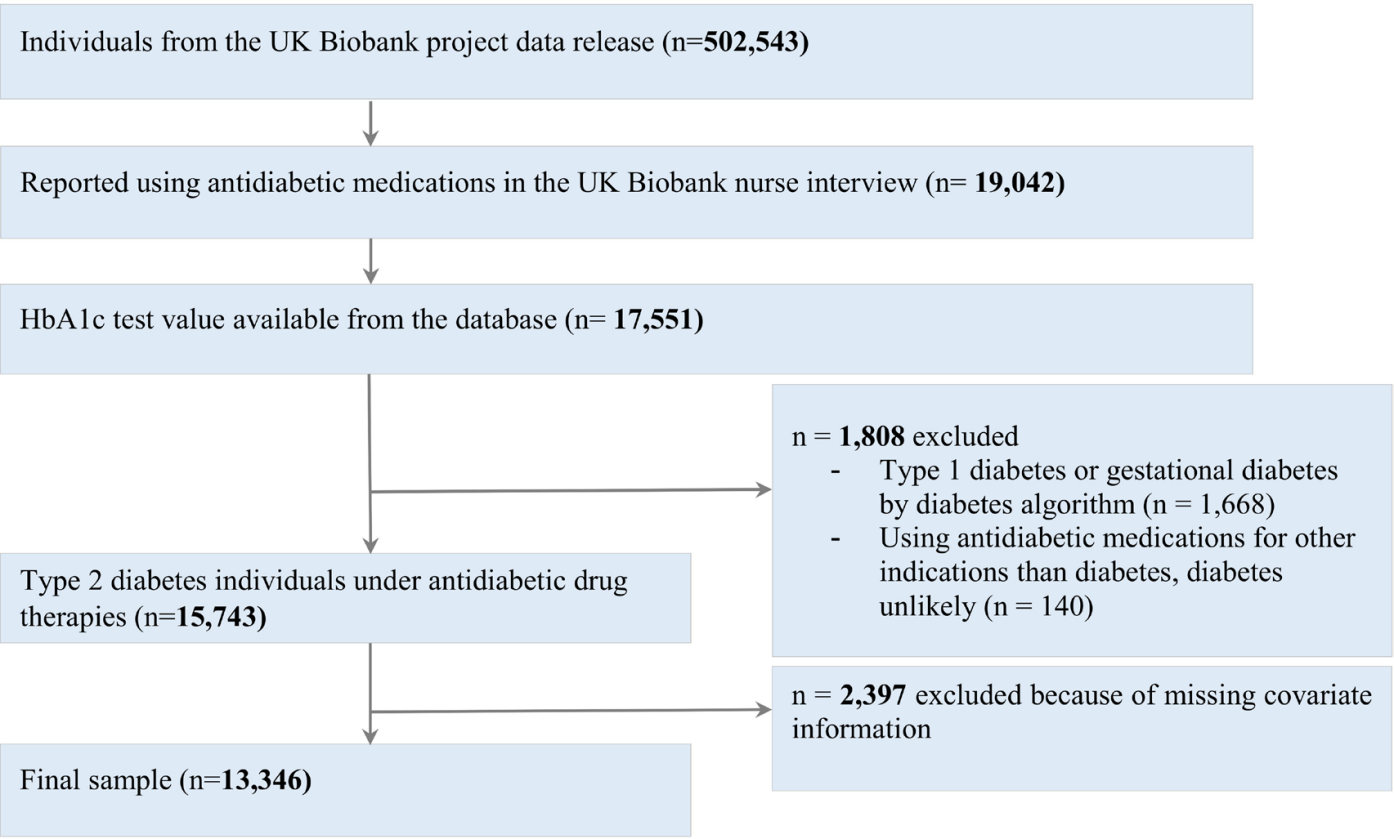
Flow diagram illustrating inclusions and exclusions to arrive at the final cohort. HbA1c, hemoglobin A1c.

The UK Biobank is a prospective population-based cohort study which enrolled over 500 000 individuals aged between 40 and 69 years. Participants were recruited from across the UK and enrolled at one of the UK Biobank assessment centers. For the present analysis, data were available from 13 346 patients with T2D on glucose-lowering medications who participated in the UK Biobank baseline investigation between 2006 and 2010 (figure 1).

Participants were classified as patients with T2D whenever one of the following criteria was met: (1) using a validated algorithm based on self-reported disease, medication, and T2D diagnosis in medical history^9^; the variables were collected by a self-completed UK Biobank touchscreen questionnaire and through a verbal interview by a trained nurse of the UK Biobank baseline assessment team; and (2) HbA1c level ≥6.5% (48 mmol/mol).^10^ Information about antidiabetic drug therapies was obtained from the treatment/medication records (UK Biobank Field ID 20003) of the UK Biobank verbal interview. Individuals with probable type 1 diabetes or probable gestational diabetes identified by the diabetes algorithm were excluded from the analysis. Participants gave informed consent.

### Assessment of sleep variables

Sleep variables were derived from the UK Biobank touchscreen questionnaire. Sleep duration (ID 1160) was measured by the question ‘About how many hours sleep do you get in every 24 hours? (please include naps)’. For the analysis, sleep duration was divided into the categories short sleep duration (6 hours or less), normal sleep duration (7–8 hours), and long sleep duration (9 hours or more). The frequency of insomnia symptoms (ID 1200) was assessed by the question ‘Do you have trouble falling asleep at night or do you wake up in the middle of the night?’. Participants had the following response options: ‘never/rarely’, *‘*sometimes’, and ‘usually’. For the analysis, the response options ‘never/rarely’ and ‘sometimes’ were merged. The risk of OSA was determined by reports of snoring (ID 1210; ‘Does your partner or a close relative or friend complain about your snoring?’; response options: ‘yes’and ‘no’), reports of daytime sleepiness (ID 1220; ‘How likely are you to doze off or fall asleep during the daytime when you don’t mean to?’; response options: ‘never/rarely’, ‘sometimes’, ‘often’, and ‘all of the time’), body mass index (BMI; ID 21001), and blood pressure (ID 4080/93 and 4079/94). Based on the scoring criteria of the Berlin Questionnaire, which is used to assess a person’s risk for OSA,^11^ the risk for OSA was considered high for UK Biobank participants when two of the three criteria were met: (1) ‘yes’ to the snoring question (ID 1210); (2) ‘often’ or ‘all of the time’ to the daytime sleepiness question (ID 1220); or (3) either obesity (BMI ≥30 kg/m^2^) or hypertension (systolic blood pressure ≥140 mm Hg or diastolic blood pressure ≥90 mm Hg).

### Outcome variable

Blood HbA1c levels (ID 30750) were centrally determined by the UK Biobank with high-performance liquid chromatography using the Bio-Rad VARIANT II TURBO HbA1c analyzer.

### Potential confounders

Potential confounders were selected based on the literature.^12–15^ Age (ID 21003), sex (ID 31), ethnic background (ID 21000), region of UK Biobank assessment center (ID 54; merged into England, Scotland and Wales), Townsend index reflecting socioeconomic status (ID 189), BMI (ID 21001), systolic blood pressure (ID 4080/93), and smoking status (ID 20116) were determined by the UK Biobank reception information, baseline characteristics, touchscreen questionnaire, or physical measurement. The level of physical activity was divided into ‘low’, ‘moderate’ and ‘high’, according to the short-form International Physical Activity Questionnaire (IPAQ) based on the total metabolic equivalent minutes per week.^16^ The short-form IPAQ questions were included in the UK Biobank touchscreen questionnaire (IDs 864, 874, 884, 894, 904, 914). The duration of T2D was calculated using the self-reported age when diabetes was first diagnosed (ID 2976) and age when the participant attended the UK Biobank assessment center (ID 21003). The therapeutic regimen of T2D was divided into monotherapy and combination therapy according to the antidiabetic medication information (ID 20003) provided in the verbal interview.

### Statistical analysis

Associations between sleep variables and HbA1c were determined by analysis of covariance (ANCOVA). The distribution of each continuous variable was visually examined by histograms. Two ANCOVA models were applied to investigate associations of sleep characteristics with HbA1c. Model 1 included participants’ sleep characteristics (ie, sleep duration, OSA, or frequency of insomnia symptoms), age, and sex. Model 2 included participants’ age, sex, ethnic background, Townsend index, BMI, systolic blood pressure, smoking status, level of physical activity, diabetes regimen, diabetes duration, sleep duration, frequency of insomnia symptoms, and risk of OSA. This model was additionally adjusted for the region of the UK Biobank assessment center. In the case of multiple comparisons (ie, when comparing the sleep duration categories with each other), post-hoc Bonferroni corrections were applied (specified in the Results section).

The dose–response association between the number of sleep problems per subject and HbA1c was examined by linear regression analysis. The number of sleep problems per subject was used to create a four-level exposure variable (0=no sleep problem; 1=high risk for OSA *OR* frequent insomnia symptoms *OR* short (≤6 hours/day) or long (≥9 hours/day) duration sleeper; 2=two sleep problems; and 3=all three sleep problems). This analysis was adjusted for participants’ age, sex, ethnic background, Townsend index, BMI, systolic blood pressure, smoking status, level of physical activity, diabetes regimen, diabetes duration age, and the region of UK Biobank assessment center.

Overall, a two-sided p value of less than 0.05 was regarded as statistically significant. Analyses were performed with Stata V.15.1.

## Results

### Cohort characteristics

The characteristics of the study population are presented in table 1. The cohort had a mean HbA1c of 7.21% (95% CI 7.19 to 7.23). Metformin (83.7% of the total population), sulfonylurea (32.9%), and insulin (20.8%) were the three most commonly used glucose-lowering medications. More than half (56.6%) of the individuals with T2D were on monotherapy. About 42% of the patients reported either short (≤6 hours/day) or long (≥9 hours/day) sleep duration. A high risk for OSA was found for 41.7% of the population. Finally, 35.8% of the patients reported frequent insomnia symptoms. The corresponding prevalence rates among non-T2D UK Biobank participants (n=488 310) were 32% for short or long sleep duration, 24% for high risk of OSA, and 28% for frequent insomnia symptoms.

**Table 1:** Characteristics of patients (N=13 346)

Characteristics	Finding*
Age, years, mean (95% CI)	60.2 (60.1 to 60.3)
Sex	
Male	8773 (65.7)
Female	4573 (34.3)
Ethnic background	
White	11 642 (87.2)
Mixed	78 (0.6)
South Asian	973 (7.3)
Black	390 (2.9)
Chinese	39 (0.3)
Others	224 (1.7)
Region of UKB assessment center	
England	11 963 (89.6)
Scotland	816 (6.1)
Wales	567 (4.3)
Townsend index, mean (95% CI)	−0.45 (−0.51 to −0.39)
BMI, kg/m^2^, mean (95% CI)	31.7 (31.6 to 31.8)
Obesity	7573 (56.7)
Systolic blood pressure, mm Hg, mean (95% CI)	143.1 (142.8 to 143.4)
Hypertension	7724 (57.9)
Smoking status	
Never	5910 (44.3)
Previous	6002 (45.0)
Current	1434 (10.7)
Level of physical activity	
Low	4757 (35.6)
Moderate	4937 (37.0)
High	3652 (27.4)
HbA1c, %, mean (95% CI)	7.21 (7.19 to 7.23)
Duration of type 2 diabetes, years, mean (95% CI)	8.2 (8.1 to 8.4)
Therapeutic regimen of type 2 diabetes	
Monotherapy	7559 (56.6)
Combination therapy	5787 (43.4)
Antidiabetic administrations	
Metformin	11 175 (83.7)
Sulfonylurea	4395 (32.9)
Glitazone	1542 (11.6)
Meglitinide	111 (0.8)
Other oral antidiabetics	46 (0.3)
Insulin	2772 (20.8)
Habitual sleep duration per day	
≤6 hours	3728 (27.9)
7–8 hours	7755 (58.1)
≥9 hours	1863 (14.0)
Snoring	6099 (45.7)
Frequent daytime sleepiness	975 (7.3)
Risk of OSA	
Low	7778 (58.3)
High	5568 (41.7)
Frequency of insomnia	
Never/rarely/sometimes	8574 (64.2)
Usually	4772 (35.8)

*Data are presented as number (percentage) of patients unless otherwise indicated.

BMI, body mass index; HbA1c, hemoglobin A1c; OSA, obstructive sleep apnea; UKB, UK Biobank.

### Sleep characteristics and HbA1c

As shown in table 2, a high risk for OSA was independently associated with higher HbA1c values (mean difference (95% CI) between patients at high risk of OSA and those at low risk of OSA: +0.07% (0.02 to 0.11), p=0.004). We also observed that self-reported long sleep duration was associated with higher HbA1c values (mean difference (95% CI) between patients reporting long sleep duration and those reporting normal sleep duration: +0.10% (0.03 to 0.18), p=0.004 (Bonferroni-corrected), as derived from the ANCOVA model 2; table 2). In the ANCOVA model 1, the mean HbA1c value in patients reporting short sleep duration was 0.08% (0.03 to 0.15) higher than those reporting normal sleep duration (p=0.002, Bonferroni-corrected). However, no significant difference in HbA1c values between short and normal duration sleepers was found in the fully adjusted ANCOVA model 2 (table 2). Finally, no differences in HbA1c between patients reporting frequent insomnia symptoms and those less regularly affected by insomnia symptoms were found (table 2).

**Table 2:** Unadjusted and adjusted mean (95% CI) HbA1c levels (%), stratified by risk of OSA, self-reported daily sleep duration, and self-reported insomnia complaints

Sleep characteristics	HbA1c (%)			
	Unadjusted	Model 1	Model 2	Model 2a
OSA risk				
Low risk (n=7778)	7.17 (7.15 to 7.20)	7.17 (7.15 to 7.20)	7.20 (7.17 to 7.23)	7.18 (7.16 to 7.21)
High risk (n=5568)	7.27 (7.24 to 7.30)	7.26 (7.23 to 7.29)	7.23 (7.20 to 7.27)	7.25 (7.22 to 7.28)
P value for main effect of OSA	<0.001	<0.001	0.22	0.004
Sleep duration				
≤6 hours (n=3728)	7.27 (7.23 to 7.31)	7.26 (7.21 to 7.29)	7.24 (7.19 to 7.27)	–
7–8 hours (n=7755)	7.17 (7.14 to 7.19)	7.17 (7.14 to 7.19)	7.18 (7.16 to 7.21)	–
≥9 hours (n=1863)	7.28 (7.23 to 7.35)	7.31 (7.26 to 7.37)	7.28 (7.23 to 7.34)	–
P value for main effect of sleep duration	<0.001	<0.001	0.003	–
Insomnia symptoms				
Never/rarely/sometimes (n=8574)	7.20 (7.17 to 7.23)	7.20 (7.17 to 7.22)	7.20 (7.17 to 7.23)	–
Usually (n=4772)	7.24 (7.20 to 7.27)	7.24 (7.20 to 7.27)	7.23 (7.19 to 7.27)	–
P value for main effect of insomnia symptoms	0.13	0.08	0.41	–

Model 1: independent variables: age, sex, sleep duration, OSA risk, and insomnia symptoms; dependent variable: HbA1c.

Model 2: independent variables: age, sex, sleep duration, OSA risk, insomnia symptoms, UK Biobank assessment center, ethnic background, Townsend index, BMI, systolic blood pressure, smoking status, level of physical activity, diabetes regimen, and diabetes duration; dependent variable: HbA1c.

Model 2a: independent variables: age, sex, sleep duration, OSA risk, insomnia symptoms, UK Biobank assessment center, ethnic background, Townsend index, smoking status, level of physical activity, diabetes regimen, and diabetes duration; dependent variable: HbA1c. BMI and systolic blood pressure were not included in model 2a as both variables were used to estimate the risk of OSA.

BMI, body mass index; HbA1c, hemoglobin A1c; OSA, obstructive sleep apnea.

**Figure 2:**
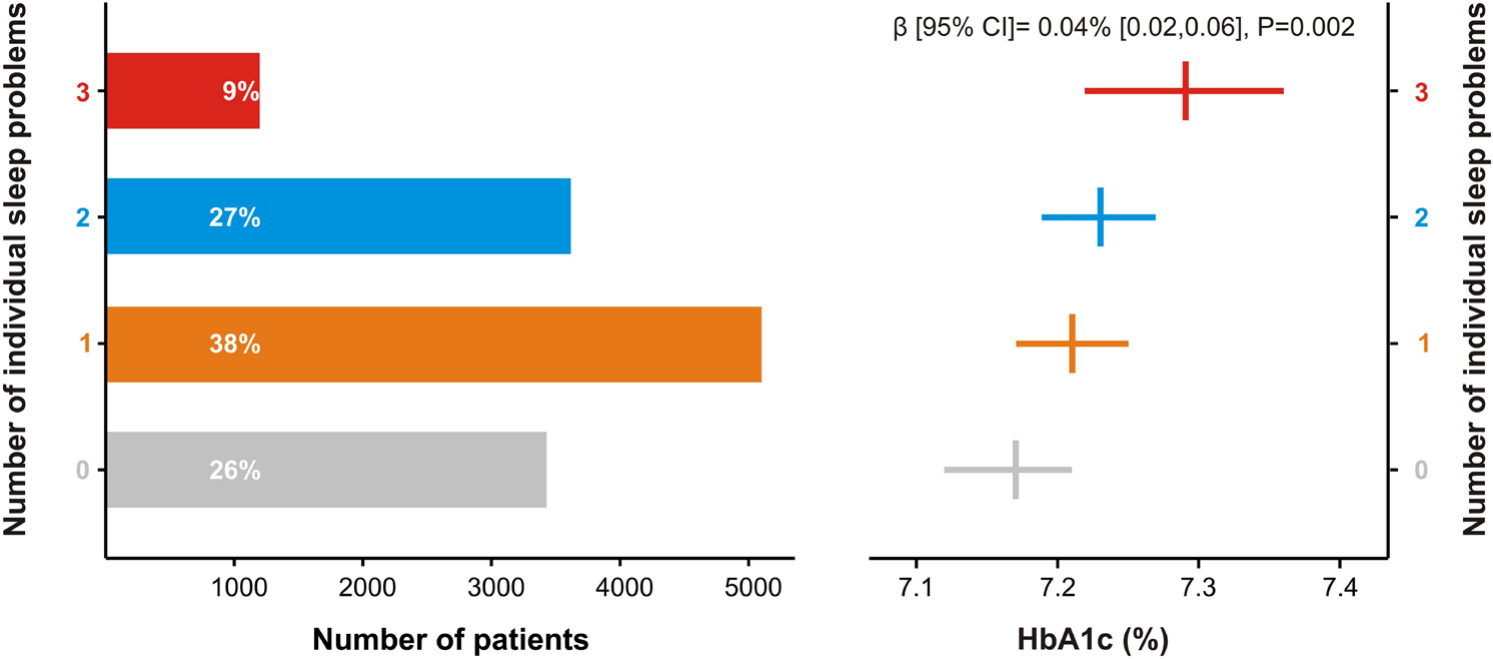
Dose–response association between the number of individual sleep problems and HbA1c. The number of sleep problems per subject was used to create a four-level exposure variable (0=no sleep problem; 1=high risk for obstructive sleep apnea *OR* frequent insomnia symptoms *OR* short (≤6 hours/day) or long (≥9 hours/day) duration sleeper; 2=two sleep problems; and 3=all three sleep problems). Mean (95% CI) HbA1c values (%) derived from analysis of covariance and were adjusted for age, sex, region of UK Biobank assessment center, ethnic background, Townsend index, body mass index, systolic blood pressure, smoking status, level of physical activity, diabetes regimen, and diabetes duration. HbA1c, hemoglobin A1c.

As suggested by the results of a separate linear regression analysis, a positive dose–response relationship was found between the number of sleep problems per patient and HbA1c (β coefficient (95% CI) 0.04% (0.02 to 0.06), p=0.002; figure 2).

## Conclusions

Using data from the UK Biobank cohort, one main finding was that long sleep duration (≥9 hours/day) was independently linked with higher HbA1c values. Poor sleep quality, sedentary lifestyle, unhealthy dietary choices, and desynchrony between circadian and behavioral states have been proposed as potential mechanisms underlying the association between long sleep duration and impaired metabolic control.^17^ We also observed a positive association between a high risk for OSA and HbA1c. OSA is characterized by hypoxia during sleep. Both acute and chronic hypoxia result in impaired glucose metabolism.^18 19^ OSA also leads to shallow and fragmented sleep, which, in addition to hypoxia, may contribute to impaired glycemic control.^20 21^

In young healthy individuals, brief episodes of curtailed or low-quality sleep have been shown to impair fasting and postprandial insulin sensitivity,^22 23^ lower glucose tolerance,^23 24^ and induce a metabolic shift from glucose toward non-glucose oxidation in insulin-dependent tissues (eg, skeletal muscle).^25 26^ In line with these findings, both short sleep duration and poor sleep quality have been linked to higher HbA1c values in observational studies among patients with T2D.^5 6 8^ In the present cross-sectional study, neither short sleep duration (defined as ≤6 hours/day) nor frequent insomnia symptoms were independently associated with higher HbA1c in the fully adjusted ANCOVA model. One explanation could be that previous studies often involved heterogeneous patient samples (eg, subjects with pre-diabetes, patients with untreated T2D, and patients with T2D on glucose-lowering medication). Our cross-sectional analysis was restricted to patients with T2D on antidiabetic drug therapy.

Using 7-day wrist actigraphy and sleep questionnaires, a previous study has investigated whether sleep characteristics may act in concert on HbA1c in patients with T2D (N=172).^4^ In combination, variability in sleep duration, total sleep duration, and subjective sleep quality was most strongly associated with HbA1c (explaining 10.3% of the variance in HbA1c).^4^ Extending these findings, in the present study, we observed a positive dose–response association between the number of sleep problems per subject and HbA1c. However, no conclusions regarding causality can be drawn due to the cross-sectional study design. It has, for instance, been shown that higher levels of HbA1c are associated with neurodegeneration.^27^ Neurodegeneration may detrimentally affect the brain regions involved in sleep/wake regulation, and as such explain the observed dose–response association. It could also be assumed that patients with multiple sleep problems may be more likely to make choices that deviate from dietary recommendations in T2D. Studies have, for instance, shown that acute episodes of sleep loss promote the intake of carbohydrate-rich foods,^28 29^ which may result in increased HbA1c values.

Whatever the underlying mechanism, the results from our study suggest that long sleep duration and a high risk for OSA are associated with higher HbA1c values in patients with T2D on glucose-lowering medications. Our results also indicate that patients with T2D suffering simultaneously from multiple sleep problems may exhibit higher HbA1c values. Thus, studies are warranted to investigate whether concomitant treatment of multiple sleep problems can meaningfully lower HbA1c among patients with T2D.

### Strength and limitations

The main strengths of the present study are its sample size and that the results were robust to adjustments. The study also had some limitations. Our results were based on a cohort of patients with T2D of mainly white British ancestry. Thus, our findings must be confirmed in T2D populations of other ethnicities. Besides, sleep variables in the present study were primarily based on self-reports. Thus, large-scale studies using objective measures of sleep, such as actigraphy (for sleep duration and sleep quality), pulse oximetry (for OSA), or sleep polysomnography, are warranted to confirm our findings. Such objective sleep measurements will also help examine the association of other sleep characteristics with HbA1c in patients with T2D (eg, depth of slow-wave sleep; sleep onset and sleep offset variability). Finally, when interpreting our results, it must be borne in mind that the associations between sleep parameters and HbA1c were small. For instance, the HbA1c of patients with T2D at high risk of OSA was about 0.07% higher than that of patients with T2D at low risk of OSA. By comparison, the glucose-lowering drug metformin typically lowers HbA1c by 1.5%–2.0% among patients with T2D.^30^ Thus, from a clinical point of view, increasing compliance with prescribed glucose-lowering medication may be a higher priority.

## References

[1] Tan X, van Egmond L, Chapman CD, et al Aiding sleep in type 2 diabetes: therapeutic considerations. Lancet Diabetes Endocrinol 2018;6:60–8. 10.1016/S2213-8587(17)30233-4 28844889

[2] Reutrakul S, Van Cauter E Interactions between sleep, circadian function, and glucose metabolism: implications for risk and severity of diabetes. Ann N Y Acad Sci 2014;1311:151–73. 10.1111/nyas.12355 24628249

[3] Aronsohn RS, Whitmore H, Van Cauter E, et al Impact of untreated obstructive sleep apnea on glucose control in type 2 diabetes. Am J Respir Crit Care Med 2010;181:507–13. 10.1164/rccm.200909-1423OC 20019340 PMC2830401

[4] Brouwer A, van Raalte DH, Rutters F, et al Sleep and HbA_1c_ in Patients With Type 2 Diabetes: Which Sleep Characteristics Matter Most? Diabetes Care 2020;43:235–43. 10.2337/dc19-0550 31719053

[5] Lee SWH, Ng KY, Chin WK The impact of sleep amount and sleep quality on glycemic control in type 2 diabetes: a systematic review and meta-analysis. Sleep Med Rev 2017;31:91–101. 10.1016/j.smrv.2016.02.001 26944909

[6] Mokhlesi B, Temple KA, Tjaden AH, et al Association of self-reported sleep and circadian measures with glycemia in adults with prediabetes or recently diagnosed untreated type 2 diabetes. Diabetes Care 2019;42:1326–32. 10.2337/dc19-0298 31048411 PMC6609965

[7] Pillai A, Warren G, Gunathilake W, et al Effects of sleep apnea severity on glycemic control in patients with type 2 diabetes prior to continuous positive airway pressure treatment. Diabetes Technol Ther 2011;13:945–9. 10.1089/dia.2011.0005 21714680

[8] Knutson KL, Ryden AM, Mander BA, et al Role of sleep duration and quality in the risk and severity of type 2 diabetes mellitus. Arch Intern Med 2006;166:1768–74. 10.1001/archinte.166.16.1768 16983057

[9] Eastwood SV, Mathur R, Atkinson M, et al Algorithms for the capture and adjudication of prevalent and incident diabetes in UK Biobank. PLoS One 2016;11:e0162388. 10.1371/journal.pone.0162388 27631769 PMC5025160

[10] International Expert Committee International expert Committee report on the role of the A1c assay in the diagnosis of diabetes. Diabetes Care 2009;32:1327–34. 10.2337/dc09-9033 19502545 PMC2699715

[11] Netzer NC, Stoohs RA, Netzer CM, et al Using the Berlin questionnaire to identify patients at risk for the sleep apnea syndrome. Ann Intern Med 1999;131:485–91. 10.7326/0003-4819-131-7-199910050-00002 10507956

[12] Li J, Chattopadhyay K, Xu M, et al Glycaemic control in type 2 diabetes patients and its predictors: a retrospective database study at a tertiary care diabetes centre in Ningbo, China. BMJ Open 2018;8:e019697. 10.1136/bmjopen-2017-019697 PMC5875602 29581203

[13] Houle J, Lauzier-Jobin F, Beaulieu M-D, et al Socioeconomic status and glycemic control in adult patients with type 2 diabetes: a mediation analysis. BMJ Open Diabetes Res Care 2016;4:e000184. 10.1136/bmjdrc-2015-000184 PMC4873951 27239316

[14] Gunton JE, Davies L, Wilmshurst E, et al Cigarette smoking affects glycemic control in diabetes. Diabetes Care 2002;25:796–7. 10.2337/diacare.25.4.796-a 11919139

[15] Rizos CV, Elisaf MS Antihypertensive drugs and glucose metabolism. World J Cardiol 2014;6:517–30. 10.4330/wjc.v6.i7.517 25068013 PMC4110601

[16] International physical activity questionnaire. Available: https://sites.google.com/site/theipaq/home [Accessed 4 May 2020].

[17] Tan X, Chapman CD, Cedernaes J, et al Association between long sleep duration and increased risk of obesity and type 2 diabetes: a review of possible mechanisms. Sleep Med Rev 2018;40:127–34. 10.1016/j.smrv.2017.11.001 29233612

[18] Oltmanns KM, Gehring H, Rudolf S, et al Hypoxia causes glucose intolerance in humans. Am J Respir Crit Care Med 2004;169:1231–7. 10.1164/rccm.200308-1200OC 15044204

[19] Hjalmarsen A, Aasebø U, Birkeland K, et al Impaired glucose tolerance in patients with chronic hypoxic pulmonary disease. Diabetes Metab 1996;22:37–42. 8697294

[20] Herzog N, Jauch-Chara K, Hyzy F, et al Selective slow wave sleep but not rapid eye movement sleep suppression impairs morning glucose tolerance in healthy men. Psychoneuroendocrinology 2013;38:2075–82. 10.1016/j.psyneuen.2013.03.018 23602132

[21] Tasali E, Leproult R, Ehrmann DA, et al Slow-Wave sleep and the risk of type 2 diabetes in humans. Proc Natl Acad Sci U S A 2008;105:1044–9. 10.1073/pnas.0706446105 18172212 PMC2242689

[22] Cedernaes J, Lampola L, Axelsson EK, et al A single night of partial sleep loss impairs fasting insulin sensitivity but does not affect cephalic phase insulin release in young men. J Sleep Res 2016;25:5–10. 10.1111/jsr.12340 26361380

[23] Wong PM, Manuck SB, DiNardo MM, et al Shorter sleep duration is associated with decreased insulin sensitivity in healthy white men. Sleep 2015;38:223–31. 10.5665/sleep.4402 25325485 PMC4288603

[24] Spiegel K, Leproult R, Van Cauter E Impact of sleep debt on metabolic and endocrine function. Lancet 1999;354:1435–9. 10.1016/S0140-6736(99)01376-8 10543671

[25] Broussard JL, Ehrmann DA, Van Cauter E, et al Impaired insulin signaling in human adipocytes after experimental sleep restriction: a randomized, crossover study. Ann Intern Med 2012;157:549–57. 10.7326/0003-4819-157-8-201210160-00005 23070488 PMC4435718

[26] Cedernaes J, Schönke M, Westholm JO, et al Acute sleep loss results in tissue-specific alterations in genome-wide DNA methylation state and metabolic fuel utilization in humans. Sci Adv 2018;4:eaar8590. 10.1126/sciadv.aar8590 30140739 PMC6105229

[27] Thomassen JQ, Tolstrup JS, Benn M, et al Type-2 diabetes and risk of dementia: observational and Mendelian randomisation studies in 1 million individuals. Epidemiol Psychiatr Sci 2020;29:e118. 10.1017/S2045796020000347 32326995 PMC7214711

[28] Spiegel K, Tasali E, Penev P, et al Brief communication: sleep curtailment in healthy young men is associated with decreased leptin levels, elevated ghrelin levels, and increased hunger and appetite. Ann Intern Med 2004;141:846–50. 10.7326/0003-4819-141-11-200412070-00008 15583226

[29] Hogenkamp PS, Nilsson E, Nilsson VC, et al Acute sleep deprivation increases portion size and affects food choice in young men. Psychoneuroendocrinology 2013;38:1668–74. 10.1016/j.psyneuen.2013.01.012 23428257

[30] Metformin – some background information. Available: https://www.diabetesnet.com/about-diabetes/diabetes-medications/metformin/ [Accessed 4 May 2020].

